# Effect of extracorporeal shock wave therapy on scar pain in burn patients: A prospective, randomized, single-blind, placebo-controlled study

**DOI:** 10.1097/MD.0000000000004575

**Published:** 2016-08-12

**Authors:** Yoon Soo Cho, So Young Joo, Huisong Cui, Sung-Rae Cho, Haejun Yim, Cheong Hoon Seo

**Affiliations:** a Department of Rehabilitation Medicine, Hangang Sacred Heart Hospital, College of Medicine, Hallym University; b Department and Research Institute of Rehabilitation Medicine, Yonsei University College of Medicine; c Department of Burn Surgery, Burn Center, Hangang Sacred Heart Hospital, College of Medicine, Hallym University, Seoul, Korea.

**Keywords:** burn, extracorporeal shock wave therapy, scar pain

## Abstract

**Background:**

Extracorporeal shock wave therapy (ESWT) has been used to reduce pain in patients with various musculoskeletal diseases and wounds. We investigated the effect of ESWT on scar pain after complete wound epithelialization in burn patients.

**Methods:**

A prospective, single-blind, placebo-controlled study was conducted from February 2014 to 2015. Forty patients with burn scar pain despite standard therapy (medication, physical therapy, and burn rehabilitation massage therapy) were randomized into ESWT or control (sham ESWT) groups. ESWT was administered at 100 impulses/cm^2^ (0.05–0.15 mJ/mm^2^) once per week for 3 weeks. The treatment effects were assessed using the numerical rating scale (NRS), pain threshold, Nirschl pain phase system, and Roles and Maudsley scores.

**Results:**

The characteristics of patients between the 2 study groups were balanced (*P* >0.05) for age, sex, and total burn surface area (%). In both groups, the NRS, pain threshold (Ib/cm^2^), and Nirschl pain phase system values significantly improved (*P* <0.05) after 3 sessions of ESWT or sham therapy, and there were significant differences between the 2 groups in terms of these 3 variables (*P* <0.001, *P* <0.001, *P* = 0.013, respectively). The Roles and Maudsley scores significantly improved; among 20 patients, 17 reported a score of poor (85%) and 3 reported fair (15%) before ESWT, whereas 3 reported poor (15%), 8 reported fair (40%), 5 reported good (25%), and 4 reported excellent (20%) after ESWT (*P* = 0.004). The scores did not improve in the control group (*P* = 0.128).

**Conclusion:**

ESWT significantly reduced scar pain in burn patients after wound recovery.

## 1 Introduction

Patients with burn injuries persistently experience severe pain exacerbated by therapeutic procedures during rehabilitation therapy and the re-epithelization process, which includes aseptic dressing and skin grafting, ^[1]^ unlike patients with skeletal muscular injuries whose pain for the treatment duration gradually subsides as they recover from morbidity, although it can progress to chronic pain. The absence of sufficient pain modulation during the treatment period for the burn injury can lead to poor compliance with physical or occupational therapy, an increased frequency of progression to chronic pain, paresthesia that persists for over a year, ^[2]^ and the persistence of depressive symptoms because of post-traumatic stress disorder (the prevalence rates vary between 8% and 45%). ^[3]^ Approximately 70% to 80% of burn patients complain of paresthetic sensation and ∼35% complain of pain in the scar tissue that lasts for more than a year.[ ^4^ ^5^] Therefore, pain modulation during the rehabilitation therapy period should be just as dynamic as it was in the initial wound treatment stage following burn injury. ^[6]^

Pain in burn patients during rehabilitation therapy is caused by a combination of direct stimulation of the skin and subcutaneous tissues that were subjected to thermal and mechanical injuries from the burn. Burn injuries over joint areas, especially in the extremities, are complicated by joint contracture as a result of long periods of immobilization for wound treatment; thus, pain modulation is very important in enabling physical therapy or occupational therapy to prevent joint contracture, and medications such as opioids, nonsteroidal anti-inflammatory drugs, and anti-psychotic drugs are presently widely used for pain modulation. ^[2]^ However, because pharmacological treatments can cause side effects and complications such as drug addiction, nonpharmacological treatments such as transcutaneous electrical nerve stimulation, ^[7]^ burn rehabilitation massage therapy, ^[8]^ and virtual reality rehabilitation system ^[9]^ are being combined in a useful manner. ^[7]^ Although various treatments such as medication ^[2]^ and the aforementioned adjuvant therapies are effective in pain modulation for burn scars during rehabilitation therapy, many burn patients still complain of pain that is not being modulated. ^[9]^ Therefore, the need for new and more effective treatments for pain modulation of burn scars has emerged.

In the early 1980s, extracorporeal shock wave therapy (ESWT) was used to destroy kidney stones. In the mid-1980s, it was used to treat musculoskeletal diseases, and in the 1990s, it was used in orthopedic procedures; presently, it has been widely used for noninvasive pain management and regenerative treatment of musculoskeletal diseases. [^10^ ^11^ ^12^ ^13^] ESWT facilitates the regeneration of damaged tendons and bones, and it relieves pain when used for diseases such as proximal plantar fasciitis, lateral epicondylitis of the elbow, patellar tendinopathy, nonunion and delayed union of long bone fractures, and avascular necrosis of the femoral head; additionally, it facilitates blood supply in patients with chronic diabetic foot ulcers or ischemic heart disease. ^[11]^ Although many clinical trials have been conducted, most studies on ESWT in burn patients have investigated its effects on wound re-epithelization in the early stage of burn injury ^[14]^ or on hypertrophic and contracture scars. ^[15]^ Therefore, on the basis of the aforementioned study results, we hypothesized that ESWT can be helpful for reducing pain by facilitating normal regeneration of injured tissues below the burnt skin in patients with pain that persists or is exacerbated after complete epithelization of the scar.

In the present study, ESWT was performed on burn patients admitted to our hospital for rehabilitation therapy following treatments for burn skin wounds at burn surgery or plastic surgery departments to evaluate the effectiveness of ESWT on residual scar pain after burn injury, and determine the clinical usefulness of ESWT as a new, noninvasive, and feasible treatment modality for scar pain.

## 2 Materials and methods

### 2.1 Study design

The present study was a prospective, randomized, single-blind, placebo-controlled study that was conducted between February 2014 and 2015 at the Burn Center of Hallym University Hangang Sacred Heart Hospital, Seoul, South Korea. The study protocol was approved by the institutional review board of Hangang Sacred Heart Hospital, and all patients who volunteered to participate in the study provided their informed consent.

### 2.2 Participants

The participants in the study included patients with complete epithelization of open skin wounds who were admitted to our Department of Rehabilitation Medicine for the first time after undergoing aseptic treatment or skin grafting at the department of burn surgery or plastic surgery at Hangang Sacred Heart Hospital. Among these patients, those who complained of burn scar pain of ≥5 points on a numerical rating scale (NRS) despite undergoing standard therapy (medication, physical therapy, and burn rehabilitation massage therapy) for ≥1 week, had tenderness on physical examination, and had pain stemming from skin and tissues damaged by burn injuries were selected. Exclusion criteria were those with scar pain caused by psychiatric disorders such as post-traumatic stress disorder and somatoform disorder; those with symptoms suggestive of fractures around the burn scar, blood clotting disorders, anti-coagulant use, or scar area infection; those with an unstable burn scar skin condition that may lead to blistering when the skin surface is stimulated during ESWT; those with a history of hyperventilation; or pregnant patients.

### 2.3 Randomization

Numbers were assigned according to the order of admission of 43 burn patients with scar pain who satisfied all of the aforementioned criteria. Then a computer program was used to divide them into the ESWT group (n = 22) and the placebo control group (n = 21). One and 2 patients in the control and ESWT groups, respectively, dropped out of the study because of transfer to another hospital; thus, 20 patients each were included in the ESWT and control groups for the final analysis (Flow Diagram).

### 2.4 Methods

Among the burn scar areas with pain, the area in which the most severe tenderness was palpated was designated as the primary treatment site, and the pain threshold (Ib/cm^2^) was measured using Algometer BASELINE^®^ (Hoggan Health Industries, Inc., West Jordan, UT). The pain threshold was defined as the mean of the pressure values at which pain is first felt when the measurements were taken twice from the area with the most severe tenderness. The measurements were taken after the patients were made aware of the fact the measuring point was the pressure at which pain is first felt and not at the point when the pain is no longer tolerable. ^[16]^

**Figure 1:**
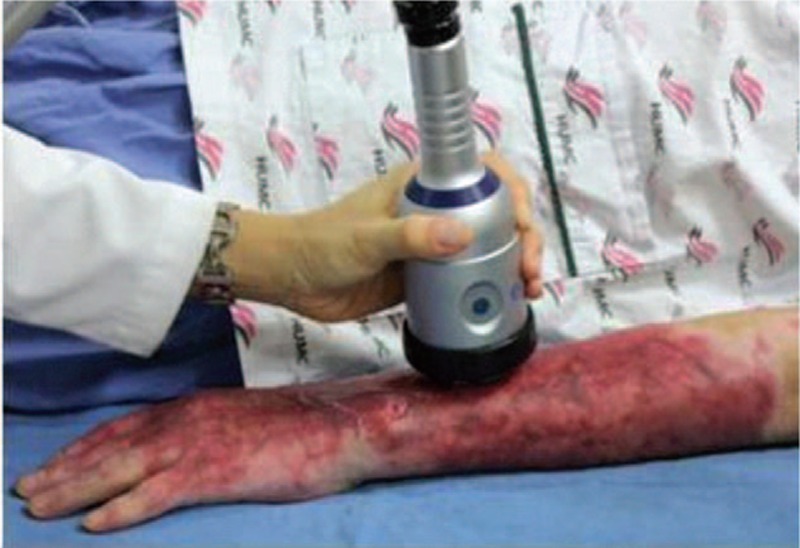
The extracorporeal shock wave therapy was administered to burn patients. The administered shock wave dose was 100 impulses/cm^2^ (depending on the burn wound surface area) at 0.05 to 0.15 mJ/mm^2^ with a total of 1000 to 2000 impulses.

ESWT was conducted using the Duolith SD-1^®^ device (Storz Medical, Tägerwilen, Switzerland) with an electromagnetic cylindrical coil source of focused shock wave (Fig. 1). Depending on the area where tenderness was palpated, shock wave therapy was performed around the primary treatment site at 100 impulses/1 cm^2^ in accordance with a previous study, ^[11]^ and depending on the patient's pain tolerance, an energy flux density (EFD) of 0.05 to 0.15 mJ/mm^2^, frequency of 4 Hz, and 1000 to 2000 impulses were administered at 1-week intervals for 3 sessions. ^[12]^ The depth of the treatment site was controlled by dividing the focal depth into 3 levels: 15, 30, and 50 mm. The shock wave focus was defined as the point within the pressure distribution model in which the pressure is ≥50% of the peak pressure, and it had a diameter of ∼20 mm. ^[17]^ Accordingly, we continued by moving the probe to tender areas in a sequential manner at a distance of 20 to 30 mm after ESWT at 100 impulses/1 cm^2^ on the primary treatment site that showed the most severe tenderness. To ensure that the shock waves were being delivered well to the target tissue, ultrasound jelly was applied between the skin and probe surfaces, after which the treatment was performed with enough pressure to enable the probe and skin to come in close contact to prevent the formation of a layer of air.

In the control group, the same shock wave equipment used in the treatment group was used with a sham adapter that had the same shape as a shock wave adapter, but it emitted no energy; thus, the patients participating in the present study, who were receiving ESWT for the first time, would not be able to distinguish whether they were being treated. From 1 week before the treatment to the final assessment, all patients received medication, physical therapy, and burn rehabilitation massage therapy for pain relief.

### 2.5 Assessment of the treatment effects

A nurse who did not know which group the patient belonged to assessed the treatment effects; the nurse performed the assessments before the first treatment and on 7th day after the 3rd treatment. An NRS was used for self-measured pain severity. This assessment method used a grading scale of 0 to 10 for pain severity, with 0 representing no pain and 10 representing unimaginably severe pain. ^[18]^

The tenderness area and pain threshold were measured using BASELINE (Hoggan Health Industries, Inc.); this is a valid and reliable method that has been extensively used for quantifying post-treatment effects in patients with soft tissue pain accompanied by tenderness. ^[16]^ The Nirschl pain phase system was used as the functional assessment of pain; it increases from phase 1 to 7 as the pain becomes more severe, with phase 1 representing cases of mild pain with exercise or pain resolved within 24 hours, and phase 7 representing cases of constant pain at rest or pain that disrupts sleep (Table 1). ^[19]^

**Table 1:**
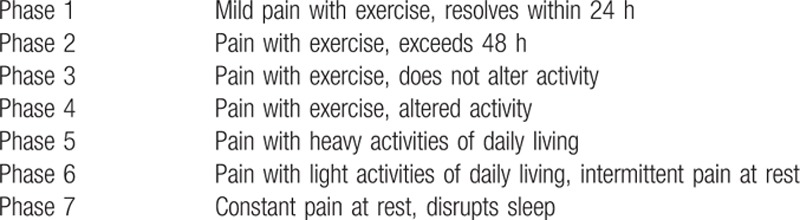
Nirschl pain phase system used for the clinical evaluation.

The Roles and Maudsley scores were used to assess the overall pain severity and final patient satisfaction with the treatment used, and they were determined depending on pain and the degree of restricted movement: ^[20]^ pain-limiting activity (poor); some discomfort after prolonged activity (fair); occasional discomfort, full movement, and full activity (good); and no pain, full movement, and full activity (excellent).

### 2.6 Statistical analysis

Collected data were analyzed using SPSS, version 18 program (SPSS Inc., Chicago, IL), with the statistical significance level set to *P* <0.05. To examine the pretreatment homogeneity between the ESWT and control groups, the Fisher exact test, Mann–Whitney test, and independent *t* test were used, whereas to examine the pretest homogeneity of the NRS and Nirschl pain phase system values, the Mann–Whitney test was performed. To examine the pretest homogeneity of the pain threshold, an independent *t* test was used, and to examine the pretest homogeneity of the Roles and Maudsley scores, the Pearson χ^2^ test was used. Moreover, to determine the pretreatment and post-treatment effects within each of the treatment and control groups, the Wilcoxon signed-rank test was used to analyze the NRS and Nirschl pain phase system values, the paired *t* test was used to analyze the pain threshold, and the Fisher exact test was used to analyze the Roles and Maudsley scores. To determine the interaction effects of the group and time (group × time) in terms of the pretreatment and post-treatment assessment results between the 2 groups, repeated-measures analysis of variance (ANOVA) was used.

## 3 Results

### 3.1 Subjects and comparison of homogeneity between the pretest assessment items

The patients’ demographics (sex ratio, age, total burn surface area, mechanism of the burn, location of the burn, and duration from the burn injury to ESWT) were similar between the 2 groups with no significant differences (*P* >0.05), which confirmed the homogeneity of the 2 groups (Table 2). An examination of the homogeneity of the pretest assessment items showed no significant differences in terms of the NRS, pain threshold, Nirschl pain phase system values, and Roles and Maudsley scores (*P* >0.05); thus, all assessment items before ESWT were also confirmed to be homogenous between the 2 groups (Table 3).

**Table 2:**
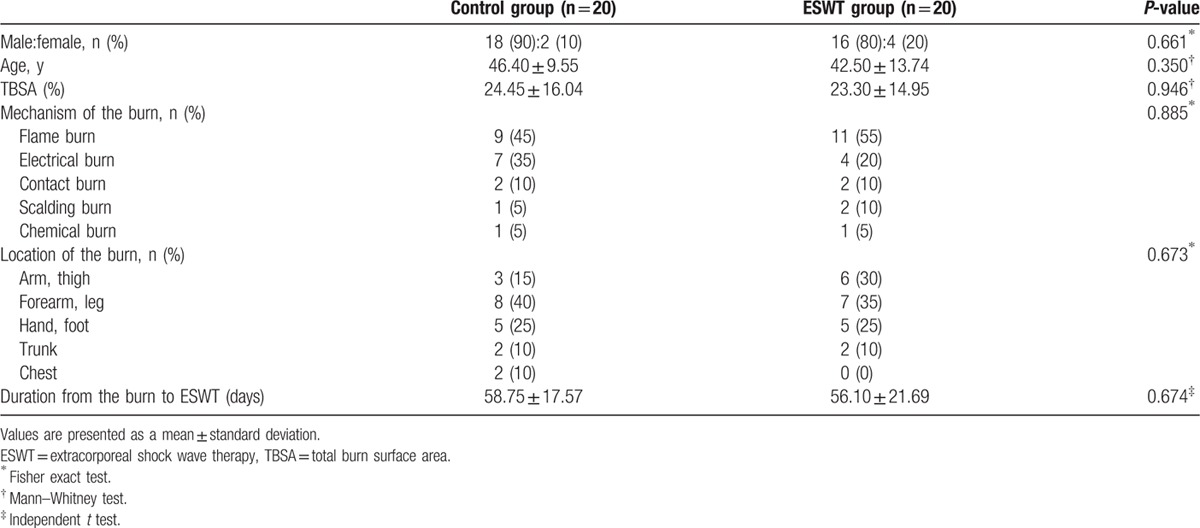
Demographic and clinical characteristics of the subjects.

**Table 3:**
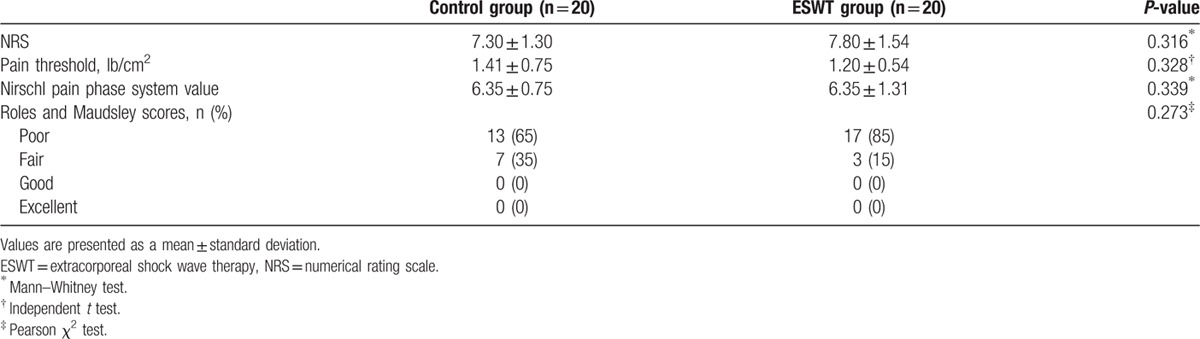
Prehomogeneity test of the preliminary assessment.

### 3.2 Changes in the NRS, pain threshold, Nirschl pain phase system values, and Roles and Maudsley scores

The NRS score significantly decreased in both groups after 3 weeks (ESWT group: 7.80 ± 1.54 to 3.80 ± 2.35 points, *P* <0.001; control group: 7.30 ± 1.30 to 5.55 ± 1.50 points, *P* <0.001). In the assessment that was performed immediately after ESWT, immediate pain reduction effects were seen in 5 patients in the ESWT group and 0 patients in the control group, with a mean score of 7.25 ± 1.585 points, which was statistically significant (*P* = 0.034). In the repeated measures ANOVA, significant interactions were seen between the group and time variables (*P* <0.001). Thus, scar pain was reduced more significantly in the group that received 3 sessions of ESWT (Table 4).

**Table 4:**
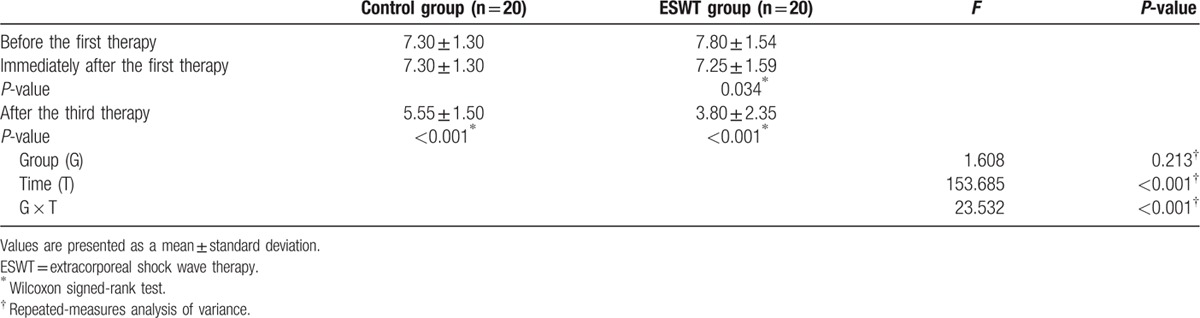
Changes in the numerical rating scale scores.

Both groups showed a statistically significant increase in the pain threshold after 3 weeks (ESWT group: 1.20 ± 0.54 to 2.60 ± 0.73 Ib/cm^2^, *P* <0.001; control group: 1.41 ± 0.75 to 2.03 ± 0.72 Ib/cm^2^, *P* <0.001). In the repeated measures ANOVA, significant interactions were seen between the group and time variables (*P* <0.001). Thus, the treatment effects were significantly better in the ESWT group than in the control group (Table 5).

**Table 5:**
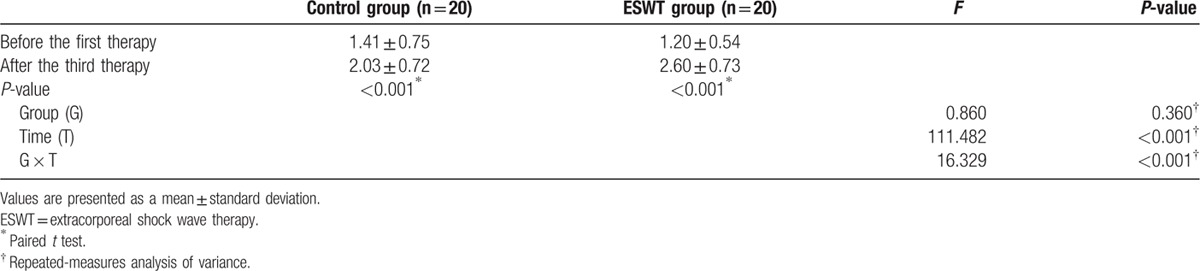
Changes in the pain threshold (Ib/cm^2^).

**Figure 2:**
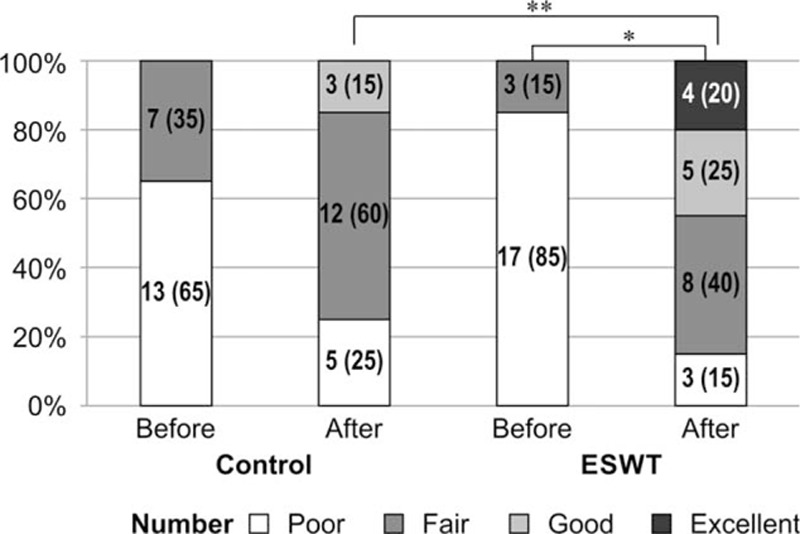
Changes in the Role and Maudsley scores. Among the 20 patients in the ESWT group, 17 (85%) reported a score of poor and 3 (15%) reported fair before ESWT, whereas 3 (15%) reported a score of poor, 8 (40%) reported fair, 5 (25%) reported good, and 4 (20%) reported excellent after ESWT. Among the 20 patients in the control group, 13 (65%) reported a score of poor and 7 (35%) reported fair before ESWT, whereas 5 (25%) reported a score of poor, 12 (60%) reported fair, and 3 (15%) reported good after sham ESWT. The Roles and Maudsley scores significantly improved after ESWT in the ESWT group (^∗^ *P* = 0.004), but they did not improve in the control group (*P* = 0.128). There were significant differences between the 2 groups (^∗∗^ *P* = 0.04). Statistical analysis was performed using the Fisher exact test. Values are presented as a number (%). ESWT = extracorporeal shock wave therapy.

Both groups showed significant reductions in the Nirschl pain phase values (ESWT group: from phase 6.35 ± 1.31 to 4.40 ± 1.82, *P* <0.001; control group: from phase 6.35 ± 0.75 to phase 5.40 ± 1.05, *P* = 0.002). In the repeated measures ANOVA, significant interactions were seen between the group and time variables (*P* = 0.013). Thus, functional improvement was more significant in the ESWT group than in the control group (Table 6). Regarding the Roles and Maudsley scores, the ESWT group showed a significant improvement after 3 treatment sessions (*P* = 0.004), whereas the control group showed no statistical difference (*P* = 0.128). Results of the Fisher exact test for the post-treatment assessments showed significant differences between the 2 groups (*P* = 0.04), which indicated that the final treatment satisfaction with regard to improvement in scar pain was significantly higher in the group that received 3 sessions of ESWT (Fig. 2).

**Table 6:**
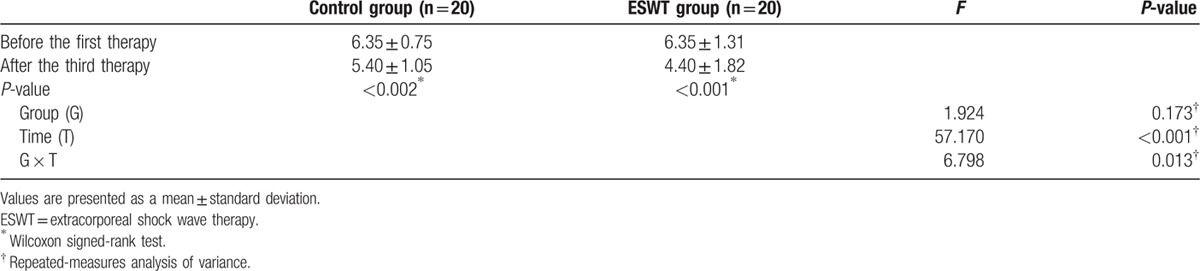
Changes in the Nirschl pain phase system values.

## 4 Discussion

The present study is the first randomized, control study to perform ESWT during rehabilitation therapy after complete epithelization of the burn wound in burn patients with scar pain that was not modulated by medication, physical therapy, or burn rehabilitation massage therapy. Previous studies have investigated the effects of ESWT on burn patients to accelerate re-epithelization ^[14]^ or treat hypertrophic and contracture scars. ^[15]^ The present study's results showed that ESWT causes a significantly greater reduction in scar pain and significantly higher patient satisfaction with regard to pain relief.

The shock wave used for ESWT has the characteristic of being a sound wave with a high positive pressure amplitude that increases very rapidly in comparison to ambient pressure. The mechanism of ESWT involves mechanotransduction, in which mechanical energy is transformed into biochemical or molecular energy in living tissues to induce a variety of specific intracellular changes, and thereby induces pain relief and tissue regeneration. It has been reported that hematological, microscopic, molecular, and immunological responses in various cells and plasma induce pain relief and tissue regeneration. ^[10]^ Increased cell membrane permeability and the destruction of damaged tissue through radical induction following ESWT have been shown to promote new tissue formation; ^[21]^ regulate secretion and synthesis of substance P; ^[22]^ decrease the number of unmyelinated nerve fibers that trigger pain; ^[23]^ increase the release of growth factors for blood vessels, epithelium, bone, and collagen; and stimulate stem cells for wound healing. ^[24]^ A study involving ESWT on patients with chronic lateral epicondylitis reported immediate pain relief following ESWT, which was attributed to the inhibition of nociceptors from repeated stimulation. ^[25]^ In the present study, 5 patients in the ESWT group showed immediate pain relief, which was statistically significant (Table 4).

The fact that the ESWT group showed significantly greater pain reduction than the control group could be attributed to the ESWT-induced vascularization, which increased blood flow to facilitate tissue regeneration and inhibit nociceptors in the peripheral skin scar to block central sensitization to pain and decrease the synthesis of substance P in the dorsal root ganglion to inhibit neuronal hyperexcitability. Both the control and ESWT groups showed significant pain reduction after 3 weeks compared with the pretreatment values, which is believed to be the result of the standard, identical regimen involving medication, physical therapy, and burn rehabilitation massage therapy that the 2 groups had received; a previous study from our laboratory demonstrated that when rehabilitation massage therapy was conducted on burn patients, the epidermal moisture evaporation rate and pain in NRS were significantly reduced. ^[8]^ The therapeutic effects of ESWT on wound regeneration are closely associated with an increase in blood flow. In a necrotic tissue transplantation model study involving artificially caused local tissue ischemia in laboratory settings, an increase in local tissue blood flow from ESWT was proven by a significant decrease in laser Doppler image signals. ^[26]^ Recent studies have indicated that nitric oxide plays the most important role in increasing local blood flow in the wound area after ESWT. Diabetic mice with diminished capacity for neovascularization and wound healing were divided into mice with an endothelial nitric oxide synthase (eNOS) genetic defect and the defect-free control group. Wounds were created artificially on the back of the mice, and the 2 groups were further divided into groups that did and did not receive ESWT. When the levels of wound recovery were assessed in all 4 groups on days 0, 3, and 10 of ESWT, the level of wound recovery and neovascular density were significantly high in the diabetic mice group without an eNOS genetic defect that received ESWT. ESWT caused endothelial shear stress, a frictional force generated on the surface of the vascular epithelium from blood flow, which acted as a powerful stimulus for the production of endothelial nitric oxide, and this increased the expression of eNOS to induce the expression of vascular endothelial growth factor, which facilitated neovascularization and enhanced wound healing. ^[12]^

Adverse effects based on the shock wave dose have been reported in animal experiments. When 100 impulses at an EFD of 0.28 mJ/mm^2^ were administered to rat Achilles tendons, transient edema and inflammation were observed, ^[27]^ and in another study when ESWT was administered to mice Achilles tendons with 2000 impulses at 0.2 mJ/mm^2^, fragmentation of collagen fibers was observed in 7 of 8 mice. ^[28]^ The histological response after ESWT is dose dependent on the applied energy (mJ); hence, when the EFD applied in the shock wave is increased, the focal range remains the same but the therapeutic range increases. ^[16]^ In the present study, ESWT was performed at 0.05 to 0.15 mJ/mm^2^ EFD at 100 impulses/cm^2^, with a total of 1000 to 2000 impulses, according to pain tolerance by the patients and the range of treatment without adverse effects.

The present study had some limitations. The sample size was small, and although the patients and evaluators were blinded to the study design, the therapist could not be blinded while conducting the treatments. Moreover, standardized treatment was not administered because no clear guidelines were available on the shock wave dose, effective time point for starting the treatment, and intervals and iterations. Furthermore, to exclude scar pain caused by psychiatric disorders, the patients were limited to those who had tenderness in the scar area; thus, we were not able to investigate the therapeutic effects of ESWT on scar pain from a variety of causes. Therefore, we believe it is necessary to determine standardized treatment guidelines by performing ESWT on more patients who have sustained burn injuries with a greater variety of scar pain at different time points.

## 5 Conclusions

Among the patients who require rehabilitation therapy after complete healing of burn wounds caused by burn surgery or plastic surgery, extracorporeal shock wave therapy (ESWT) was performed on those who, despite conventional treatments, had persistent pain considered to have stemmed from soft tissue below the scar. Overall, the ESWT group demonstrated significantly greater pain reduction than the control group.

Therefore, we think that ESWT can be an effective alternative treatment for decreasing scar pain in burn patients, and future studies should evaluate the shock wave dose, treatment time, and interval and frequency with a greater number of patients and at various time points.

## Acknowledgments

We appreciate Dong Hyun Kim, PhD, Professor in the Department of Social and Preventive Medicine, Hallym University, for teaching Dr. Cho how to use theoretical and practical statistics for the purpose of this research study. This was a 2015 master's thesis for the School of Medicine at Hallym University, South Korea.
